# Features of glycemic variations in drug naïve type 2 diabetic patients with different HbA_1c_ values

**DOI:** 10.1038/s41598-017-01719-y

**Published:** 2017-05-08

**Authors:** Feng-fei Li, Bing-li Liu, Reng-na Yan, Hong-hong Zhu, Pei-hua Zhou, Hui-qin Li, Xiao-fei Su, Jin-dan Wu, Dan-feng Zhang, Lei Ye, Jian-hua Ma

**Affiliations:** 1 Department of Endocrinology, Nanjing First Hospital, Nanjing Medical University, Nanjing, China; 2 National Heart Research Institute Singapore, National Heart Centre, Singapore, Singapore

## Abstract

To define the features of glycemic variations in drug naïve type 2 diabetic (T2D) patients with different HbA_1c_ values using continuous glucose monitoring (CGM), a total of 195 drug naïve T2D patients were admitted. The subjects were divided into the following groups: lower HbA_1c_ values (≤8%), moderate HbA_1c_ values (>8% and ≤10%), and higher HbA_1c_ values (>10%). The patients underwent oral glucose tolerance tests and were then subjected to 3-day CGM. The primary endpoint was the differences in the 24-hr mean amplitude of glycemic excursions (MAGE) in patients with different HbA_1c_ values. Patients with higher HbA_1c_ values had larger MAGEs than those in the moderate and lower groups (7.44 ± 3.00 vs. 6.30 ± 2.38, P < 0.05, 7.44 ± 3.00 vs. 5.20 ± 2.35, P < 0.01, respectively). The 24-hr mean glucose concentrations increased incrementally in the patients with lower, moderate and higher HbA_1c_ values. Moreover, the patients with higher HbA_1c_ values exhibited higher peak glucose concentrations and prolongation in the time to peak glucose. Patients with higher HbA_1c_ values had larger MAGE compared with those with lower and moderate HbA_1c_ values. Our data indicated patients with higher HbA_1c_ values should receive special therapy aimed at reducing the larger glycemic variations.

## Introduction

Large glucose fluctuations in patients with may have implications for the risk for long-term diabetic complications^1, 2^. The underlying mechanisms might be the acute glucose fluctuations, or more specifically, the triggering the oxidative stress by acutely increased postprandial blood glucose levels^3^.

Postprandial glucose is an independent risk factor for cardiovascular disease^4^. Acute glucose fluctuations during postprandial periods other than chronic hyperglycemia have been shown to play an important role in oxidative stress in patients with type 2 diabetes (T2D)^3^. Microvascular and macrovascular complications are mainly^5, 6^, or at least partially^6, 7^, dependent on hyperglycemia. The rapid rise in postprandial blood glucose concentrations induce an over-production of peroxynitrite and nitrotyrosine^3, 8, 9^. Continued efforts have been made to suppress postprandial hyperglycemia in patients with T2D^10^. Research has indicated that improved postprandial excursions could smooth the oxidative and nitrosative stress^11^.

HbA_1c_ is very useful as evidence of long term improvement in mean glucose in the large scale clinical studies of the treatment of T2D^12–14^. Reductions in HbA_1c_ value in patients with diabetes leads to a reduction in the risk of death, myocardial infarction, and microvascular complications^7^. HbA_1c_ does not necessarily reflect daily plasma glucose fluctuations; however, as different glucose profiles can confer similar HbA1c values, patients with similar HbA_1c_ values do not necessarily bear the same glycemic variations^1, 2, 15^. Therefore, glycemic fluctuations should be considered when constructing strategies aimed to reduce the burden of diabetic complications as well as HbA1c values^2^. Continuous Glucose Monitoring (CGM) provides a unique opportunity to examine the 24-hrs glucose excursions in patients with T2D.

We therefore performed a single-center, open and retrospective study. In this study, we determined 24-hr glycemic variations using CGM in drug naïve T2D patients with different or even similar HbA_1c_ values.

## Results

A total of 195 drug naïve T2D patients who met the inclusion criteria (129 men and 66 women, aged 51.06 ± 9.87 years, BMI 25.08 ± 2.98 kg/m², and HbA_1c_ values 9.32 ± 1.62%) were recruited into the current study.

The patients were then divided into three groups according to HbA_1c_ values (L group with HbA_1c_ values ≤8%, M group with HbA_1c_ values >8% and ≤10%, and H group with HbA_1c_ values >10%). A total of 55, 90 and 50 subjects were allocated into the L, M and H groups with mean HbA_1c_ values of 7.60 ± 0.32%, 9.13 ± 0.53% and 11.55 ± 1.36%, respectively. We observed that the BMI in the H group was significantly lower than that in the L group (24.19 ± 3.11 vs. 25.47 ± 2.91, P < 0.05). Moreover, patients in the H and M groups were younger than those in the L group (48.40 ± 9.08 vs. 54.09 ± 9.73, P < 0.01, 50.68 ± 9.98 vs. 54.09 ± 9.73, P < 0.05, respectively) (Table 1).

**Table 1:** Baseline for the study subjects.

Items	L Group	M Group	H Group
N (Male/Female)	55 (37/18)	90 (56/34)	50 (36/14)
Age (Yrs)	54.09 ± 9.73	50.68 ± 9.98^a*^	48.40 ± 9.08^a**^
BMI	25.47 ± 2.91	25.34 ± 2.88	24.19 ± 3.11^a*^
HbA1c (%)	7.60 ± 0.32	9.13 ± 0.53	11.55 ± 1.36
Bg 0 (mmol/L)	8.53 ± 1.66	10.09 ± 1.78^a**^	12.40 ± 2.24^ab**^
Bg 30 (mmol/L)	14.69 ± 2.46	15.59 ± 2.43	18.16 ± 3.41^ab**^
Bg 120 (mmol/L)	18.47 ± 3.43	21.50 ± 3.75^a*^	24.51 ± 4.58^ab*^
Cp 0 (pmol/L)	2.58 ± 0.90	2.42 ± 0.82	1.81 ± 0.58^ab*^
Cp 30 (pmol/L)	4.40 ± 2.15	3.54 ± 1.59^a*^	2.50 ± 1.11^ab*^
Cp 120 (pmol/L)	8.10 ± 2.51	5.74 ± 2.02^a*^	3.72 ± 1.52^ab*^
Ins 0 (mU/L)	8.97 ± 4.85	8.06 ± 4.75^a*^	4.84 ± 2.26^ab*^
Ins 30 (mU/L)	24.07 ± 17.25	18.04 ± 15.69	8.44 ± 4.87^ab*^
Ins 120 (mU/L)	48.50 ± 26.40	28.03 ± 17.63^a*^	14.72 ± 10.08^ab*^
HOMA-IR	3.42 ± 1.87	3.69 ± 2.35	2.58 ± 1.15^ab*^
Matsuda Index	85.04 ± 75.47	94.63 ± 71.63	125.88 ± 59.67^ab*^
HOMA-B	37.81 ± 23.88	25.46 ± 15.90^a**^	12.01 ± 6.81^ab**^

Data are presented as the means ± SD. L group: Lower HbA_1c_ values group, M group: Moderate HbA_1c_ values group, H group: higher HbA_1c_ values group, ^a*^compared with the L Group (*P* < 0.05), ^a**^compared with M Group (*P* < 0.01), ^b*^compared with M Group (*P* < 0.05), ^b**^compared with M Group (*P* < 0.01), Bg 0: blood glucose concentrations before glucose loading, Bg 30: blood glucose concentrations at 30 min after glucose loading, Bg 120: blood glucose concentrations at 120 min after glucose loading, Cp 0: serum C-Peptide concentrations before glucose loading, Cp 30: serum C-Peptide concentrations 30 min after glucose loading, Cp 120: serum C-Peptide concentrations 120 min after glucose loading, Ins 0: serum insulin concentrations before glucose loading, Ins 30: serum insulin concentrations 30 min after glucose loading, Ins 120: serum insulin concentrations 120 min after glucose loading.

Oral glucose tolerance test (OGTT) data showed that patients with higher HbA_1c_ values exhibited higher blood glucose concentrations and lower C-peptide and insulin levels at 0, 30, and 120 min after glucose load (Table 2).

**Table 2:** CGM monitored blood profiles in study subjects.

Items	L Group	M Group	H Group
24-hrs MG (mmol/L)	9.46 ± 1.85	11.47 ± 2.19^a**^	13.52 ± 2.20^ab**^
SDMG (mmol/L)	2.00 ± 0.79	2.55 ± 0.90^a**^	2.88 ± 0.99^a**b*^
MAGE (mmol/L)	5.20 ± 2.35	6.30 ± 2.38^a*^	7.44 ± 3.00^a**b*^
AUC (<3.9 mmol/L*Day)	0.01 ± 0.07	0.00 ± 0.01	0.00 ± 0.03
AUC (>10.0 mmol/L*Day)	0.80 ± 0.93	2.18 ± 1.57^a**^	3.76 ± 1.90^ab**^
PG (mmol/L)	13.23 ± 3.49	16.27 ± 3.62^a**^	18.63 ± 2.98 ^a**b**^
Time to peak (min)	91.36 ± 31.01	82.94 ± 26.54	108.00 ± 30.19^ab*^

Data are presented as the means ± SD. L Group: Lower HbA_1c_ values group, M Group: Moderate HbA_1c_ values group, H Group: higher HbA_1c_ values group, ^a*^compared with the L Group (*P* < 0.05), ^a**^compared with M Group (*P* < 0.01), ^b*^compared with M Group (*P* < 0.05), ^b**^compared with M Group (*P* <  < 0.01), ^ab**^compared with the L and M Groups (P < 0.01), ^ab*^compared with the L and M Groups (P < 0.05), 24-hr MG: 24-hr mean glucose concentrations, SDMG: 24-hr standard deviation of MG, AUC: the incremental area under the curve, PG: peak glucose concentrations.

CGM data showed that 24-hr mean glucose concentrations (MG) (11.47 ± 2.19 vs. 9.46 ± 1.85 mmol/L, P < 0.01; 13.52 ± 2.20 vs. 9.46 ± 1.85 mmol/L, P < 0.01; 13.52 ± 2.20 vs. 11.47 ± 2.19 mmol/L, P < 0.01, respectively); the 24-hr standard deviation of the MG (SDMG) (2.55 ± 0.90 vs. 2.00 ± 0.79 mmol/L, P < 0.01; 2.88 ± 0.99 vs. 2.00 ± 0.79 mmol/L, P < 0.01; 2.88 ± 0.99 vs. 2.55 ± 0.90 mmol/L, P < 0.05, respectively); the 24-hr mean amplitude of glycemic excursions (MAGE) (6.30 ± 2.38 vs. 5.20 ± 2.35 mmol/L, P < 0.05; 7.44 ± 3.00 vs. 5.20 ± 2.35 mmol/L, P < 0.01; 7.44 ± 3.00 vs. 6.30 ± 2.38 mmol/L, P < 0.05, respectively), and the incremental area under the curve (AUC) of the glucose above 10 mmol/L (2.18 ± 1.57 vs. 0.80 ± 0.93 mmol/L*Day, P < 0.01; 3.76 ± 1.90 vs. 0.80 ± 0.93 mmol/L*Day, P < 0.01; 3.76 ± 1.90 vs. 2.18 ± 1.57 mmol/L*Day, P < 0.01, respectively) were progressively and significantly increased alongside HbA1c values in drug naïve patients with T2D. There were no differences in the incremental AUC less than 3.9 mmol/L between the L, M and H groups (0.007 ± 0.07 vs. 0.009 ± 0.01 mmol/L*Day, P > 0.05; 0.007 ± 0.07 vs. 0.002 ± 0.003 mmol/L*Day, P > 0.05; 0.009 ± 0.01 vs. 0.002 ± 0.03 mmol/L*Day, P > 0.05, respectively) (Table 2). We also compared glycemic variations in subjects with HbA_1c_ values from 8% to 10%, although there were no differences in the MAGE, the 24-hr MG, the incremental AUC above 10 mmol/L or less than 3.9 mmol/L, or the SDBG between the two subgroups (HbA_1c_ values >8 and <9%, and ≥9 and <10%), with the exception of patients with HbA_1c_ values ≥ 9 and <10%, who had higher peak glucose concentrations than those with HbA_1c_ values ≥ 8 and <9% (Supplementary Table 1). In agreement with the similar glycemic variations, the hourly mean plasma glucose concentrations did not differ between the two groups, with the exception of the 24-hr mean glucose concentrations (Fig. 2).

**Figure 1:**
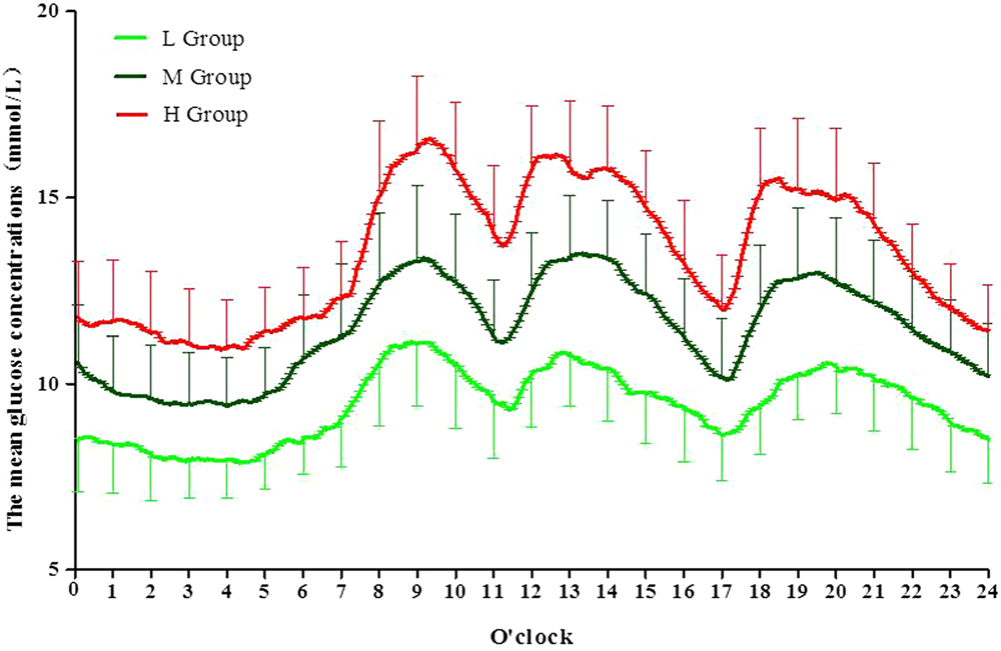
The average glucose concentrations per hour in the L, M, and H groups.

**Figure 2:**
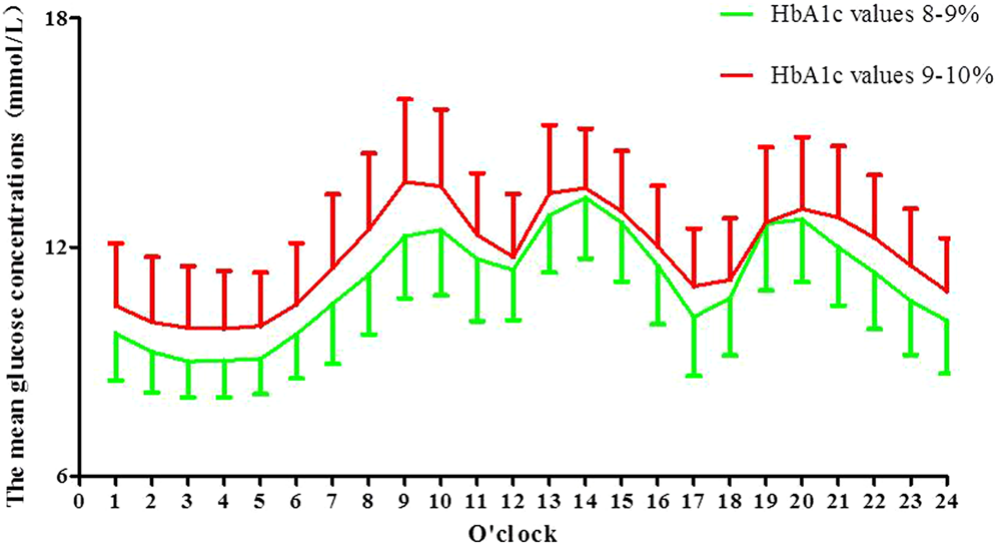
The average glucose concentrations per hour in patients with HbA_1c_ values from 8% to 10%.

The average blood glucose concentrations per hour in the H group were higher than in the M group. As expected, the average blood glucose concentrations per hour in the M group were also significantly higher than those in the L group (Fig. 1). Moreover, higher peak glucose concentrations and prolongation in the time to glucose peak after breakfast were observed in the H group. Subjects in the H group exhibited higher peak glucose concentrations than in the L and M groups (18.63 ± 2.98 vs. 13.23 ± 3.49 mmol/L, P < 0.01, 18.63 ± 2.98 vs. 16.27 ± 3.62 mmol/L, P < 0.01, respectively). In accordance with the increased peak glucose concentrations, the time to peak in patients with higher HbA1c values was significantly prolonged compared with subjects in the L and M groups (108.00 ± 30.19 vs. 91.36 ± 31.01 min, P < 0.05, 108.00 ± 30.19 vs. 82.94 ± 26.54 min, P < 0.01, respectively). However, prolongation of glucose time to peak after lunch and dinner were not observed in the current study.

We also used HbA1c as a continuous rather than a discrete variable. Multiple linear regression analyses were performed to assess the independent effects (MBG, SDMG, MAGE, FBG, PBG, HOMA-B, and HOMA-IR) on HbA1c. Our data showed that only FBG, MBG, HOMA-IR and SDBG remained significant in the stepwise regression analysis. The standardized regression coefficients were 0.476 (t = 5.735, P < 0.01), 0.260 (t = 3.016, P < 0.01), −0.197 (t = −3.370, P < 0.05) and 0.150 (t = 2.444, P < 0.05), respectively.

We also observed the contributions of SDMG and MAGE to HbA1c values. The patients were further divided into three equal groups according to the tertiles of SDBG or MAGE. The HbA1c values gradually increased from the lowest to the highest tertile of SDBG or MAGE (8.69 ± 1.35 vs. 9.37 ± 1.49 vs. 9.90 ± 1.77 mmol/L, all P < 0.01, and 8.64 ± 1.27 vs. 9.57 ± 1.60 vs. 9.75 ± 1.73 mmol/L, all P < 0.01). Our data showed that both SDMG and MAGE were strongly correlated with HbA1c values (r = 0.361, P < 0.01, and r = 0.319, P < 0.01, respectively).

We also analyzed β-cell function and insulin sensitivity in patients with different HbA_1c_ values. The multivariate analysis controlled for age and BMI to determine the significance of the differences between groups, particularly with respect to the insulin sensitivity/resistance. Our data showed that, as expected, patients with higher HbA1c values had lower HOMA-B values (H group vs. M group vs. L group: 12.01 ± 6.81 vs. 25.46 ± 15.90 vs. 37.81 ± 23.88, P < 0.01, respectively). Interestingly, patients with higher HbA1c values (>10%) had significant induction of Matsuda index (125.88 ± 59.67 vs. 94.63 ± 71.63, P < 0.05, 125.88 ± 59.67 vs. 85.04 ± 75.47, P < 0.05, respectively) compared to those in the M and L groups. In accordance with the increased Matsuda index, the HOMA-IR in patients with higher HbA1c values was significantly decreased compared to patients in the M group (2.58 ± 1.15 vs. 3.69 ± 2.35, P < 0.05), and was insignificantly reduced compared with those in the L group (2.58 ± 1.15 vs. 3.42 ± 1.87, P > 0.05). We did not observe a difference in HOMA-IR between the M and L groups (Table 1).

## Discussion

This relatively large study revealed a novel observation that glycemic variations gradually increased with HbA_1c_ values in drug naïve T2D patients. We also observed that drug naïve patients with HbA_1c_ values above 10% exhibited larger blood glucose fluctuations, higher peak glucose concentrations, and prolongation in the glucose time to peak after breakfast. In addition, patients with moderate HbA_1c_ values (>8% and ≤10%) had similar glycemic variations, with the exception of 24-hrs mean blood glucose. Our data indicated that patients with higher HbA_1c_ values should receive “special therapy” aimed at reducing the larger glycemic variations and the increased peak glucose concentrations.

A achievement of the optimal HbA_1c_ target in patients with T2D is the main consideration for physicians when choosing glucose lowering therapy^16^. HbA_1C_ is generated by the exposure of overall blood profiles for an extended period, which does not necessarily reflect daily plasma glucose variations throughout the day^1, 2^. CGM provides a unique opportunity to examine the 24-hr glycemic excursions in patients with T2D, which might be a better tool to determine overall blood glucose profiles. CGM shows the potential effectiveness in subjects with diabetes^17^ and in patients using intensive insulin therapy^18^. A set of metrics obtained from CGM could be used to describe the glycemic variations (GV) in type 1 and type 2 diabetes^19–21^. Furthermore, an observational study reported that prediabetic obese subjects have higher GV, namely SD and MAGE, compared with normal weight individuals^22^. Studies have demonstrated that there was a high degree of correlation between SD and MAGE^17, 23^. The percent coefficient of variation (%CV) displays the interpretation of GV^17, 23, 24^. Using CGM data, clinical researchers and clinicians could efficiently evaluate the quality of the glycemic control, which might be important for decision-making^25^.

In the current study, our CGM data revealed that patients with different HbA_1c_ values exhibited different glycemic variations. Diabetic patients with higher HbA_1c_ values had larger MAGE, larger SDMG, and increased incremental AUC >10.0 mmol/L, Moreover, patients with higher HbA_1c_ values exhibited increased 24-hr MG. Interestingly, patients with HbA_1c_ values above 10% had higher peak glucose concentrations and a prolongation in the glucose time to peak after breakfast. We could infer that the reason that the patients who had higher HbA1c values exhibited larger glycemic variations might partially depend on the higher peak glucose concentrations and prolongation of glucose time to peak. However, our data could not address the underlying mechanisms of the increase in peak glucose concentrations and the prolongation of the glucose time to peak.

In the current study, the decreased HOMA-IR and increased Matsuda index in the H group compared with those in the L group indicated that the patients with HbA_1c_ values above 10% had lower insulin resistance. Moreover, we also observed that the BMI in the H group was significantly lower than that in the L group. One speculation might be that these patients had very poor glycemic control, and they therefore had suffered weight loss from the urinary loss of calories. The Chinese have lower BMI and smaller waist circumferences when compared to the Western participants who had higher BMI and waist circumferences^26^. In addition, Chinese patients also have a higher percentage of body fat than Europeans and African Americans at the same level of BMI^27, 28^. Thus, we could infer that decreased body weight might be the reason for the increased insulin sensitivity, because body fat weight is associated with decreased Matsuda index and increased HOMA-IR^29^. The increased insulin sensitivity in patients with HbA_1c_ values above 10% compared with patients with HbA_1c_ values less than 10% indicated that HbA_1c_ values might correlate with the Matsuda index and HOMA-IR values. HOMA-IR in patients with higher HbA_1c_ values was significantly reduced compared with the values of those in the M group (2.58 ± 1.15 *vs*. 3.69 ± 2.35, P < 0.05). The difference in HOMA-IR was not evident between the L and M group. This finding might be attributed to the fact that we used only 138 out of 195 serum samples collected from patients at 0, 30, and 120 min after glucose loading, which were used to measure the glucose, insulin, and C-peptide concentrations. Future studies using HbA_1c_ values are needed to identify changes in insulin resistance. Our data also suggested that, as expected, patients with higher HbA_1c_ values had lower pancreatic β cell function (HOMA-B values).

A stratified analysis comparing the blood glycemic profiles in patients with moderate HbA_1c_ values, from 8% to 10% revealed that each increase of 1% in HbA_1c_ value did not result in any significant differences in hourly blood glucose concentrations, peak glucose concentrations, or blood glycemic variations (Supplementary Table 1).

The current study described a novel observation of gradually increased glycemic variations with HbA_1c_ values in a stepwise manner in drug naïve T2D patients, and patients with higher HbA_1c_ values (>10%) had higher peak glucose concentrations and prolonged glucose time to peak after breakfast. These results differ from those of a previous study that reporting the longer glucose time to peak was observed after breakfast and dinner in drug-naïve, Japanese type 2 diabetic patients with higher HbA_1c_ values^30^. They observed that the Peak Time and the Increase Range were maximal after dinner^30^. However, in the current study, the maximal blood glucose increase range was only observed after breakfast, this might be due to the different number of study subjects recruited. Future studies are needed to identify changes in blood variations in drug naïve type 2 diabetic patients with different HbA_1c_ values.

In the current study, we observed that drug naïve diabetic patients with higher HbA_1c_ values (>10%) may require receive more attention in order to address the larger blood glycemic variations, the higher glucose peak concentrations, as well as the prolongation of glucose time to peak. However, the study patient population was limited to the Nanjing area in China; therefore, the situation might not be the same for other geographical regions or populations. Evidence has demonstrated that patients with T2D in China are quite variable when compared to Western countries, such as the thrifty gene, which is prevalent in the Chinese^31^, the different pattern of intake of nutrients and life-style^32^, the lower insulin dose requirements, and the higher remission rate following short intensive insulin therapy^33^. Moreover, Asian T2D populations have the lower BMI and smaller waist circumferences compared to the Western participants^26^. The “special therapy” recommended in this study might include acarbose^34^, DPP-4 inhibitor^35^, GLP-1 receptor agonists^36^, and SGLT-2 inhibitor therapy^37^. These therapies have shown potential improvements in glucose fluctuation^34–37^ and lower insulin dose requirement by patients with T2D to maintain euglycemic control in Chinese populations^35, 36^. Furthermore, these “special therapies” should be given to patients with high MAGE and HbA_1c_ values over 10%. Our study has other limitations. First, the patient population was very heterogeneous (with A1C value from 6 to 12%). Second, untreated patients were mainly varying in the duration and severity of their diabetes, with differing beta cell reserve, and differing insulin sensitivities.

In conclusion, our data reveal that the glycemic variations gradually increased with the HbA_1c_ values in drug naïve T2D patients. Our data also indicated that patients with higher HbA_1c_ values might need some special therapies aimed at reducing the larger glycemic variations and the prolongation in time-to-peak hyperglycemia, especially after breakfast.

## Methods

This was a single-center, open and retrospective trial. Between June 2010 and November 2015, a total of 195 drug naive T2D patients were recruited in Nanjing First Hospital, Nanjing Medical University, China. The inclusion criteria were 1) Patients aged between 18 and 80 years; 2) Newly diagnosed, drug-naive T2D patients; 3) BMI 21 to 35 kg/m^2^. Patients were excluded if they had ketoacidosis, chronic kidney disease, positive antiglutamic acid decarboxylase (aGAD) antibodies, or if they had maturity onset diabetes in the young (MODY) or mitochondria diabetes mellitus^38^. Patients with known cancers were excluded^38, 39^. The study was approved by the ethics committee of Nanjing First Hospital, Nanjing Medical University. All patients gave written informed consent. The methods were conducted in accordance with the Declaration of Helsinki guidelines, including any relevant details.

Recruited patients were admitted as inpatients. Serum samples were obtained at 0, 30, and 120 min after oral administration of 75 g glucose for HbA_1c_, glucose, insulin and C-peptide concentrations determination. Plasma insulin was determined using an insulin radioimmunoassay kit (Beijing Technology Company, Beijing, China). HbA_1c_ was measured by a DiaSTAT HbA_1c_ analyzer (Bio-Rad, Hercules, CA). C-peptide and glucose concentrations were measured centrally at the central laboratory in Nanjing First Hospital, Nanjing Medical University. After the baseline parameters were assessed, retrospective CGM (Sof-sensor, CGMS-Gold, Medtronic Incorporated, Northridge, USA) was performed for 3 days, as described previously^34, 40^. Briefly, the CGM sensor was subcutaneously embedded on Day 0 approximately 16:00–17:00 PM. The patients continued with the sensors, if CGM was going well. Subjects were instructed to keep the sensor fixed and waterproof. The study nurse inputted at least 4 calibration readings every day. On Day 4, at approximately 16:00–17:00 PM, subjects had the sensors removed, and the CGM data were saved by the investigator, as described previously^34, 40, 41^. All subjects were instructed to maintain physical activity according to their doctors’ personalized instructions and received meals consisting of a total daily caloric intake of 25 kcal/kg/day. The percentages of carbohydrate, proteins and fats were 55%, 17% and 28%, respectively. Patients were instructed to have breakfast, lunch and dinner at 0700, 1100 and 1700, respectively. Subjects were then divided into a lower HbA_1c_ values (≤8%) group (L group), a moderate HbA_1c_ values (>8% and ≤10%) group (M group), and a higher HbA_1c_ values (>10%) group (H group).

The 24-hr MG, the SDBG, and the incremental AUC of blood glucose above 10.0 mmol/L or less than 3.9 mmol/L, and the hourly MG were calculated by software provided by Medtronic Incorporated, USA. The MAGE was calculated manually for each patient by measuring the arithmetic mean of the ascending and descending excursions between consecutive peaks and nadirs for the same 24-hr period, and only absolute excursion values >1 SD were considered, as described previously^34, 40^. The glucose time to peak after breakfast was also calculated among groups. β-cell function was assessed by the homoeostasis model assessment B (HOMA-B), the insulin sensitivity was indicated by HOMA-IR^38, 42^ and the Matsuda index was calculated as previous described^29, 43^.

The primary endpoint was the difference in MAGE in patients with different HbA_1c_ values. Secondary endpoints were the differences in 24-hr MG, SDBG, incremental AUC of blood glucose above 10.0 mmol/L or less than 3.9 mmol/L, peak glucose concentrations and glucose time to peak after breakfast, hourly MG, β-cell function and insulin resistance among patients with different HbA_1c_ values.

### Statistical analysis

All data are presented as the means ± SD. Statistical significance was determined by one-way analysis of variance (ANOVA), or two-way ANOVA for repeated measurements for the group comparisons, followed by Bonferroni-Dunn post hoc test. P < 0.05 was considered to be statistically significant. We used correlation coefficients and multiple linear regressions analyses to examine the interrelationships among the glycemic variations and HbA1c. All of the statistical analyses were performed using the Statistical Product and Services Solutions (SPSS) package (Version 11.5, SPSS, Science, Chicago, USA).

## Electronic supplementary material

Dataset 1
