# Supplementary material for: Features of glycemic variations in drug naïve type 2 diabetic patients with different HbA1c values
Source: Sci Rep. 2017 May 8;7:1583. doi: 10.1038/s41598-017-01719-y (PMC5431480; doi:10.1038/s41598-017-01719-y)
Supplement: Supplementary file 1 — Dataset 1 [file 41598_2017_1719_MOESM1_ESM.doc]

**Features of glycemic variations in drug naïve type 2 diabetic patients with different HbA_1c_ values**

**Author names:** Feng-fei Li, PhD.,^1^* Bing-li Liu, PhD.,^1^* Reng-na Yan, PhD.,^1^* Hong-hong Zhu, M.D.,^1^* Pei-hua Zhou, M.D.,^1^* Hui-qin Li, M.D.,^1^ Xiao-fei Su, M.D.,^1^ Jin-dan Wu, M.D.,^1^ Dan-feng Zhang, M.D.,^1^ Lei Ye, M.D., PhD.,^2^ and Jian-hua Ma, M.D., PhD.^1^**^**^**

**Author affiliations:**

^1^Department of Endocrinology, Nanjing First Hospital, Nanjing Medical University, Nanjing, China

^2^National Heart Research Institute Singapore, National Heart Centre Singapore, Singapore

**Supplementary Table 1. CGM monitored blood profiles in study subjects**

| Items | Group 1 | Group 2 | P value |
| --- | --- | --- | --- |
| 24-hrs MG (mmol/L)  SDMG (mmol/L)  MAGE (mmol/L) | 11.04±1.95  2.45±0.79  6.08±2.10 | 11.82±2.34  2.63±0.97  6.47±2.61 | 0.09  0.33  0.44 |
| AUC (<3.9 mmol/L*Day) | 0.00±0.00 | 0.00±0.02 | 0.20 |
| AUC (>10.0 mmol/L*Day) | 1.82±1.36 | 2.47±1.68 | 0.05 |
| PG (mmol/L) | 15.29±3.42 | 17.10±3.61 | 0.02 |
| Time to peak (min) | 83.29±26.57 | 82.65±26.79 | 0.91 |

Data were presented as means ± SD. Group 1: HbA_1c_ values ≥8 and <9% group, Group 2: HbA_1c_ values ≥9 and <10% Group, 24-hrs MG: 24-hrs mean glucose, SDMG: 24-hrs standard deviation of MG, AUC: the incremental area under curve, PG: peak glucose concentrations.
